# Molecular surveillance for drug-resistant *Plasmodium falciparum* in clinical and subclinical populations from three border regions of Burma/Myanmar: cross-sectional data and a systematic review of resistance studies

**DOI:** 10.1186/1475-2875-11-333

**Published:** 2012-09-19

**Authors:** Tyler Brown, Linda S Smith, Eh Kalu Shwe Oo, Kum Shawng, Thomas J Lee, David Sullivan, Chris Beyrer, Adam K Richards

**Affiliations:** 1 Johns Hopkins University School of Medicine, Broadway Research Building, 733 N. Broadway, Suite 147, Baltimore, MD, 21205, USA; 2 Global Health Access Program, 2550 Ninth Street, Ste 111, Berkeley, CA, 94710, USA; 3 Karen Department of Health and Welfare, PO Box 189, Mae Sot, Tak, 63110, Thailand; 4 Office of the Director of the Health Department, Kachin Baptist Convention 135/Shan Su (South), Myitkyina, Kachin State, Myanmar; 5 Department of Molecular Microbiology and Immunology, Johns Hopkins Bloomberg School of Public Health 615 North Wolfe St, Room E5628, Baltimore, MD, 21205, USA; 6 School of Medicine, University of California at Los Angeles, 924 Westwood Blvd, Suite 300, Los Angeles, CA, 90024, USA; 7 Department of Epidemiology Johns Hopkins Bloomberg School of Public Health, 615 N. Wolfe St., Suite E7152, Baltimore, MD, 21205, USA; 8 Department of General Internal Medicine and Health Services Research, University of California at Los Angeles, 911 Broxton Ave, Los Angeles, CA, 90025, USA

**Keywords:** Malaria, Plasmodium falciparum, Artemisinin resistance, Genetic, Subclinical infection, Conflict, Myanmar

## Abstract

**Background:**

Confirmation of artemisinin-delayed parasite clearance in *Plasmodium falciparum* along the Thai-Myanmar border has inspired a global response to contain and monitor drug resistance to avert the disastrous consequences of a potential spread to Africa. However, resistance data from Myanmar are sparse, particularly from high-risk areas where limited health services and decades of displacement create conditions for resistance to spread. Subclinical infections may represent an important reservoir for resistance genes that confer a fitness disadvantage relative to wild-type alleles. This study estimates the prevalence of resistance genotypes in three previously unstudied remote populations in Myanmar and tests the *a priori* hypothesis that resistance gene prevalence would be higher among isolates collected from subclinical infections than isolates collected from febrile clinical patients. A systematic review of resistance studies is provided for context.

**Methods:**

Community health workers in Karen and Kachin States and an area spanning the Indo-Myanmar border collected dried blood spots from 988 febrile clinical patients and 4,591 villagers with subclinical infection participating in routine prevalence surveys. Samples positive for *P. falciparum* 18 s ribosomal RNA by real-time PCR were genotyped for *P. falciparum* multidrug resistance protein (*pfmdr1)* copy number and the *pfcrt* K76T polymorphism using multiplex real-time PCR.

**Results:**

*Pfmdr1* copy number increase and the *pfcrt* K76 polymorphism were determined for 173 and 269 isolates, respectively. Mean *pfmdr1* copy number was 1.2 (range: 0.7 to 3.7). *Pfmdr1* copy number increase was present in 17.5%, 9.6% and 11.1% of isolates from Karen and Kachin States and the Indo-Myanmar border, respectively. *Pfmdr1* amplification was more prevalent in subclinical isolates (20.3%) than clinical isolates (6.4%, odds ratio 3.7, 95% confidence interval 1.1 - 12.5). P*fcrt* K76T prevalence ranged from 90-100%.

**Conclusions:**

Community health workers can contribute to molecular surveillance of drug resistance in remote areas of Myanmar. Marginal and displaced populations under-represented among previous resistance investigations can and should be included in resistance surveillance efforts, particularly once genetic markers of artemisinin-delayed parasite clearance are identified. Subclinical infections may contribute to the epidemiology of drug resistance, but determination of gene amplification from desiccated filter samples requires further validation when DNA concentration is low.

## Background

Genetically determined artemisinin-delayed parasite clearance or tolerance, first documented on the Thai-Cambodia border, has emerged on the border of Thailand and Myanmar [1,2]. Recently published estimates of malaria mortality trends suggest that the previous spread of chloroquine (CQ) and sulphadoxine-pyrimethamine resistance from Southeast Asia to Africa [3,4] contributed to the large increase in malaria mortality from 1980–2004 [5], and spread of delayed parasite clearance to Africa would represent a global health catastrophe [6].

In recognition of Myanmar’s central location between Southeast Asia and Africa, the WHO outlined a country-specific strategy for Myanmar Artemisinin Resistance Containment (MARC) [7]. The MARC surveillance strategy calls for therapeutic efficacy studies and day-3, parasite-positivity monitoring of artemisinin combination therapy (ACT) in over 20 locations. However, a strategy is lacking to assess drug resistance in remote populations of Myanmar that government and international NGO services have difficulty reaching.

Although molecular markers of artemisinin resistance or delayed parasite clearance have yet to be identified, tracking markers of resistance to partner drugs provides a valuable tool to inform coformulation policy and monitor progress of the Global Plan for Artemisinin Containment (GPARC) and MARC. Clinical and parasitological failure after treatment with an ACT is partially determined by the local efficacy of non-artemisinin partner drugs [8]. Furthermore, the decreased parasiticidal effect of artemisinin along the Myanmar-Thai border places greater reliance on partner drugs. Tracking *Plasmodium falciparum* multidrug resistance protein (*pfmdr1*) gene copy number (CN) has become an important surveillance tool, particularly in populations receiving artesunate-mefloquine. *Pfmdr1* CN is associated with delayed response to ACT, including artesunate-mefloquine [9] and artemether–lumefantrine [10,11], as well as resistance to multiple monotherapy including mefloquine [12-15].

Molecular strategies are particularly valuable for surveillance in remote, displaced and conflict-affected populations facing security and logistical constraints that make conventional *in vivo* or *in vitro* resistance studies impractical [16]. Village health workers (VHWs) trained by local community-based organizations play a key role delivering malaria control services in hard-to-access areas of Myanmar [17-19] emerging from decades of conflict, but community based organizations and VHWs have not contributed to past resistance surveillance efforts. Recent studies successfully estimated *pfmdr1* (CN) using blood samples collected on filter paper [20], and this simplified protocol makes it possible to extend the quantitative assessment of gene copy number to remote settings lacking the capacity for storage and transport of whole or fractionated blood products.

One question of potential importance in resistance containment is the relative contribution of asymptomatic persons to the reservoir of genetic resistance. Asymptomatic infections provide a parasite reservoir that contributes to malaria transmission even in areas of low or unstable transmission intensity [21-26], but uncertainty exists regarding the role of asymptomatic infections in the transmission of genetic changes conferring drug-resistance. Genetic markers of drug resistance in *P. falciparum,* including *pfcrt* K76T haplotypes and *pfmdr1* amplification, [27] are associated with decreased parasite fitness and impaired within-host growth [28]. Less fit parasites may be more likely to produce low parasitemia infections that remain asymptomatic. Persistent carriage of resistant parasites in asymptomatic populations is unlikely in situations where multiclonal infections are common, given that more fit parasites with a single copy of *pfmdr1* are likely to out-compete less fit parasites with multiple copies of *pfmdr1*. However, asymptomatic carriage could be favoured in relatively low transmission settings such as those included in this study where host immunity may be insufficient to clear infection and mono-infection with a single resistant clone may be common, particularly if de-amplification of *pfmdr1* CN is rare. In addition, compensatory mutations that decrease the fitness cost of *pfmdr1* amplification may allow drug-resistant clones to persist during multiclonal infections. The persistence of drug-resistant infections [29], including multicopy *pfmdr1* infections [30], may also be favoured by higher gametocyte carriage that appears to increase their transmission potential relative to drug-sensitive, wild-type infections.

This study sought to assess the feasibility of estimating the prevalence of elevated *pfmdr1* CN and the *pfcrt* K76T allele in three previously unstudied remote or conflict-affected populations living along Myanmar’s borders with India, China and Thailand. This study investigated the contribution of subclinical infections to the epidemiology of genetic resistance by testing the *a priori* hypothesis that resistance gene prevalence would be higher among isolates collected during active population screening compared to isolates collected from febrile clinical patients. The findings have been situated in context by conducting a systematic review of *in vivo*, *in vitro*, and molecular resistance studies in Myanmar and border regions of neighbouring countries. Resistance study locations were mapped relative to areas of recent civil conflict to test the second *a priori* hypothesis that politically unstable areas would be under-represented among resistance studies in Myanmar.

## Methods

### Study sites and participants

**Figure 1:**
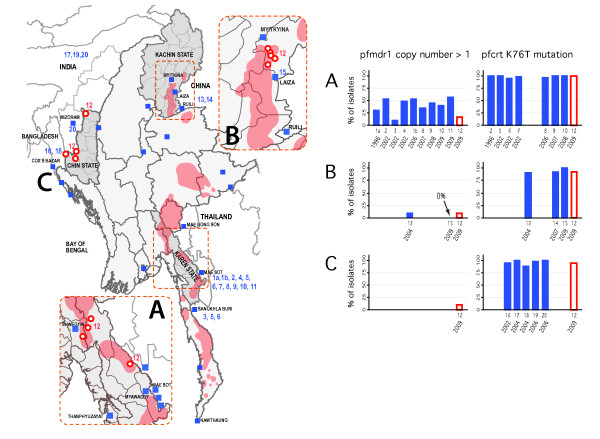
**Anti-malarial, drug-resistance studies in Myanmar and neighbouring countries, by region and year.** Blue squares represent the locations of *in vivo*, *in vitro* and molecular studies conducted between 1996 and 2009; locations of the current study (2008–2009) appear as red circles (n = 13 sites). Studies reporting prevalence of *pfmdr1* copy number amplification or *pfcrt* K76T haplotypes are numbered on the map and on the x-axis of the bar figures, in order of data collection year. Previous molecular studies from Myanmar appear in insets; molecular studies from neighbouring countries appear on the country-wide map. Locations of villages forcibly displaced between 2008–2011 are indicated by red shading. See manuscript text for additional information on displaced villages, and Additional file 1 for a complete list of molecular studies and abstracted prevalence estimates.

Between February 2009 and January 2010, three community based organizations established 13 surveillance sites in three remote regions of Myanmar: Karen State, Kachin state, and an area spanning the border of Chin State, Myanmar and Mizoram State, India (Figure 1). Populations were selected based on history of malaria burden, feasibility of sample collection, and past exclusion from other surveillance programmes due to inaccessibility or security concerns. Samples were collected by community health workers (CHWs) and VHWs who extend the reach of community-based malaria control services to unstable areas, as previously described [18]. The red-shaded areas of the map in Figure 1 indicate the locations of villages displaced since 2008 in Karen and Kachin States, where armed conflict was ongoing or imminent, respectively. Sites in border areas of Chin State were located in areas where exposure to human rights violations were widespread [31].

Oral, informed consent was obtained from all participants. Ethical approval for the study was obtained from relevant authorities at each community based organization; Johns Hopkins School of Public Health Committee on Human Subjects approved secondary analysis of the data.

Samples were collected during routine malaria programme activities modified to assess the prevalence of non-*P. falciparum* species using PCR and validate the use of a *P. falciparum*-specific, histidine-rich protein 2 (HRP-II) based rapid diagnostic test (RDT, Paracheck®, Orchid Biomedical Systems, Goa, India) in the setting of clinical diagnosis and population-based screening. CHWs collected blood spots from consecutive clinic patients with self-reported fever and from a sub-sample of villagers participating in routine bi-annual community malaria prevalence surveys [18,19,32]. All participants were tested for *P. falciparum* using the RDT and had a blood sample collected on Whatman 903 Protein Saver® cards. The sampling protocol for the programme evaluation was designed to yield approximately n = 540 *Pf-*positive samples: n = 80 from febrile clinical patients and n = 100 from villagers participating in active screenings in each of three regions. Based on a real-time polymerase chain reaction (RT-PCR) budget sufficient to test 2,100 samples, the target sample number for each region was set at 700 (500 screening and 200 clinical). Because historical programme data indicated that *P. falciparum* population prevalence in each region was consistently below 15-20%, the number of screening participants was increased to yield approximately 100 *P. falciparum* -positive isolates. False-positive RDT results due to persistent HRP-II antigenaemia were expected to approximately offset false-negative RDT results due to low sensitivity in the setting of low parasitaemia infection.

With an expected RDT-positivity rate of approximately 40%, sample collection was anticipated to yield ~80 *P. falciparum* -positive isolates from 200 febrile clinical patients in each region. When preliminary results suggested a lower RDT positivity rate (18-33%), the period of sample collection was extended from two to six months. PCR was performed on all RDT-positive samples and a region-specific fraction of RDT-negative samples from screening participants (22-34%) and clinic patients (17%-70%) sufficient to yield approximately 500 + 200 = 700 samples for RT-PCR from each region.

### DNA extraction and *Plasmodium falciparum* detection by real-time PCR

Following an existing protocol [33], DNA was extracted from dried blood samples using a commercial, 96-well, DNA extraction kit from Promega (Madison, Wisconsin, USA). For each sample, DNA from three 5-mm punches (equivalent to approximately 25 μL whole blood) was extracted and concentrated four-fold by glycogen-acetate precipitation. *Plasmodium falciparum* DNA was identified using a validated TaqMan multiplex real-time PCR assay for *P. falciparum* 18 s ribosomal DNA [33], a molecular method for *P. falciparum* detection that has sensitivity and specificity comparable to nested PCR protocols [34,35]. Isolates were considered *P. falciparum-*positive if 18 s PCR end-cycle fluorescence exceeded 300 RFU. All PCR reactions were run in 384-well plates on the Bio-Rad CFX384 Real-Time System (Bio-Rad, Hercules, CA, USA). Primers and probes for TaqMan PCR were synthesized by Integrated DNA Technologies (Coraville, Iowa, USA).

### *pfmdr1* copy number determination by multiplex real-time PCR

*pfmdr1* copy number was determined using a modification of previous assays [13,36]. Primers and a FAM-labelled probe specific for *pfmdr1*, plus primers and a Texas Red-labelled probe specific for *P. falciparum* β-tubulin*,* were amplified as a multiplex. Each reaction included β-tubulin primers (100nM), β-tubulin probes (100nM), *pfmdr1* primers (300nM), *pfmdr1* probes (200nM), Bio-Rad iQ Multiplex Powermix (1x), and 5 μL DNA, in a total reaction volume of 10 ul.

Amplification curves were analysed and cycle threshold (Ct) values determined using Bio-Rad CFX Manager software. *pfmdr1* copy number was calculated using the predictive efficiency method [36], which corrects the ΔΔ Ct method for differences in amplification efficiency between the reference gene and gene of interest. Genomic DNA from *P. falciparum* strains 3D7, D10, Dd2 were included as internal controls on each 384-well plate (MR4, Virginia, USA). These strains are known to have one, one, and three to four *pfmdr1* copies, respectively. Genomic DNA from 7C424, a strain previously determined to have two copies of *pfmdr1*, was also used to validate the assay. Mean copy number estimates were 1.15 for D10 (SD = 0.20, 32 repeats), 2.31 for 7C424 (SD = 0.42, 35 repeats), and 2.98 for Dd2 (SD = 0.39, 35 repeats).

All reactions were run in triplicate and individual replicates were rejected if they did not display exponential kinetics. The precision of *pfmdr1* CN estimates was calculated for a given isolate as the ratio of the range (maximum–minimum) divided by the mean of isolate-specific replicates (n = 2 or 3). Copy number estimates were repeated if the difference between replicates was greater than 50% of the mean estimate, or if the average cycle threshold number for *Pf* β-tubulin was greater than 34. CN estimates were considered invalid if the variance remained greater than 50% of the mean estimate or Ct remained greater than 34 after the third repeat.

In the course of the study, a higher proportion of subclinical screening isolates than clinical isolates produced invalid CN estimates. A *post-hoc* analysis explored the reasons for this discrepancy and assessed the technical feasibility of using filter paper blood samples to estimate *pfmdr1* gene copy number in isolates from a predominantly asymptomatic population with relatively low peripheral blood parasite concentrations. Recent evidence suggests that the volume of blood available from filter-paper samples may be insufficient to achieve highly accurate PCR results, particularly when parasitaemia level is low [26]. Current methods to assess gene copy number may be particularly sensitive to DNA concentration, because copy number is a function (ratio) of two quantitative amplification thresholds. Past studies assessed *pfmdr1* CN using filter paper-based samples collected from febrile clinical patients [20]; this study explored the feasibility of extending these methods to predominantly asymptomatic infections among active screening participants, and whether the variance in CN estimates was associated with parasite DNA concentration. The precision of *pfmdr1* CN estimates was plotted against cycle threshold (Ct) values, a proxy for parasitaemia level. Ct values are inversely proportional to the log DNA concentration in a given isolate, and a three-cycle increase in the Ct corresponds to approximately a 10-fold decrease in relative concentration of *P. falciparum* DNA.

### Detection of *pfcrt* polymorphisms by multiplex real-time PCR

*Plasmodium falciparum*-positive samples were analysed for wild-type *pfcrt* and *pfcrt* alleles carrying the K76T polymorphism, using validated primers and probes for the CVIET and CVMNK haplotypes [37]. Each PCR reaction included forward and reverse primers for the *pfcrt* gene (200nM), a Texas Red-labelled probe specific for the wild-type allele (200nM), a FAM-labelled probe specific for the K76T allele (200nM), Bio-Rad iQ Multiplex Powermix (1x), and 5 μL DNA, in a total reaction volume of 10 μl. Average fluoresence of the last five PCR cycles (“end-cycle fluoresence”) was used to determine the presence or absence of the wild-type and K76T polymorphisms. Genomic DNA from the CQ-sensitive D10 and CQ-resistant Dd2 strains were included on each 384-well plate as references. All samples were run in duplicate and criteria for identifying CQ-sensitive parasites were chosen for high specificity. Samples were identified as carrying the wild-type CQ-sensitive allele if both replicates had end-cycle fluorescence greater than 5% of the range between negative and positive controls.

#### Statistical analysis

Data was entered into an ACCESS database using range and consistency checks. Statistical analyses were performed using Stata 12.1. Standard errors were corrected for clustering of the data by village cluster (n = 13).

#### Systematic review of *Plasmodium falciparum* anti-malarial resistance studies in Burma/Myanmar and its border regions

PubMed and Google Scholar were searched for clinical, *in vitro*, and molecular studies evaluating resistance of *Plasmodium falciparum* to CQ, MQ and ACTs in Myanmar and neighbouring countries. Search terms included “falciparum”, “resistance”, “efficacy”, “Burma”, “Myanmar”, “Thailand”, “China”, “India”, “Bangladesh”, “pfmdr1”, “pfcrt”, “chloroquine”, “mefloquine”, “lumefantrine”, “artemisinin combination therapy”, “anti-malarial”; and minor variations on these terms. This search generated 860 publications. Reference lists of studies identified in the primary search were searched, as was data from public sources, including WHO Southeast Asia Region and country-specific publications of the WHO and respective Ministries of Health. The review included reports from Bangladesh, India and China if *P. falciparum* isolates were collected from areas bordering Myanmar, and restricted the review to reports published in English after 1995. Only observational and clinical intervention studies and related review articles were included; studies that reported only basic science or methods-oriented findings were excluded. Eighty-two manuscripts met these inclusion criteria. Reports were reviewed for clinical efficacy (failure rates, recrudescence/re-infection, parasite clearance time), *in vitro* susceptibility data (i e, IC50s) and molecular markers associated with resistance (*pfmdr1* SNPs, *pfmdr1* amplification, and the K76T *pfcrt* mutation) to CQ and MQ monotherapy, and any ACT. Figure 1 shows locations of all studies meeting criteria for this review (blue squares); molecular studies are numbered for reference to the adjacent bar graphs and are listed in Additional file 1. Resistance study locations were determined from published manuscripts and by contacting study authors. Unstable areas of Karen and Kachin States were identified based on evidence of forced displacement of villages since 2008 provided by the Thai-Burma Border Consortium [38] and a community-based organization providing humanitarian assistance in Kachin State (Figure 1).

## Results

Active screening of 4,591 out of 14,982 people living in 13 village-clusters identified 157 isolates positive for *P. falciparum* 18 s DNA, and sequential testing of 988 febrile clinical patients identified 133 *P. falciparum*-positive isolates (Table 1).

**Table 1:** Study population characteristics

	**Study Region**								
	**Karen**	**Kachin**	**Chin**						
**Number of study sites**	4	5	4						
Population	6176	4170	4636	(Total population: 14,982)					
					Percentage of study participants % (number)				
Study participants^b^	Karen	Kachin	Chin	Total study participants		RDT result available^a^	*Pf* positive by RDT	Tested by PCR	*Pf* positive by PCR
Age <5 years	459	284	201	17% (944)		99% (938)	13% (121)	34% (327)	5% (49)
5-15 years	717	662	522	33% (1,901)		99% (1,881)	15% (281)	36% (684)	7% (138)
>15 years	996	947	873	50% (2,816)		97% (2,752)	13% (371)	34% (964)	4% (99)
Female	1,152	1118	801	54% (3,071)		99% (3,038)	12% (375)	35% (1,070)	4% (136)
Asymptomatic screening participants	1,900	1,639	1,178	80% (4,717)		97% (4,591)	9% (430)	32% (1,507)	3% (157)
Febrile clinical patients	536	254	427	20% (1,217)		81% (988)	35% (343)	42% (506)	11% (133)
Total participants	2,436	1,893	1,605	5,934		94% (5,579)	14% (773)	34% (2013)	5% (290)

*RDT* rapid diagnostic test; *PCR* polymerase chain reaction; *Pf Plasmodium falciparum*.

^a^ The second round of clinic treatment books in Karen sites were not available to link RDT results to filter-paper blood spots (n = 228). All filter samples were eligible for selection for PCR.

^b^ Cells do not add to 5,934 due to missing age and sex data; percentages may not add to 100% due to rounding.

### pfmdr1 copy number

**Figure 2:**
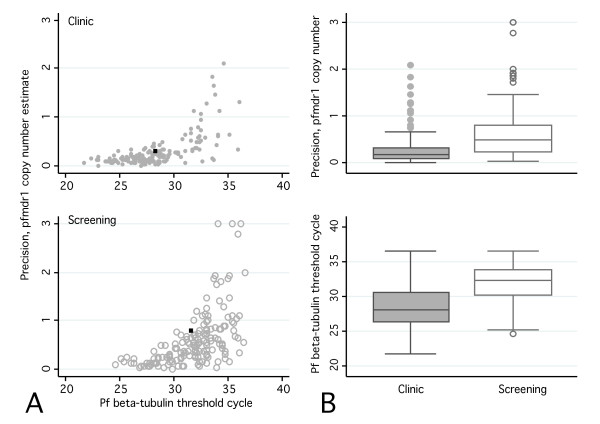
**Scatterplots and boxplots of *pfmdr1 *copy number estimate precision and *Plasmodium falciparum *beta-tubulin PCR cycle thresholds (Ct) for febrile clinical patients (grey circles and boxplots) and active screening participants (empty circles and boxplots) A:** The scatterplots demonstrate precision of *pfmdr1* copy number (CN) estimate (y-axis) diminishes as DNA concentration (x-axis) decreases among active screening participants (bottom panel) and febrile clinical patients (top panel). The mean cycle threshold and mean precision of *pfmdr1* CN is indicated by the black point on each scatterplot. A three-cycle increase in the Ct corresponds to a 10-fold decrease in relative concentration of *Pf* DNA. *Pfmdr1* precision is calculated as the range/mean for each isolate, with higher values indicating lower precision. The standard deviation of *pfmdr1* CN estimates was 0.200 for clinic patients and .516 for screening participants (not shown) **B:** Boxplots display the distribution of DNA concentration (top panel) and *pfmdr1* CN precision. Boxes represent inter-quartile ranges (IQR); whiskers represent the value of [upper/lower quartile +/− (IQR*1.5)]. Screening participants had lower DNA concentration and less precise estimation of *pfmdr1* CN.

Of 290 *P. falciparum-*positive isolates, 173 (59.6%) were successfully genotyped for *pfmdr1* copy number (Table 2). Thirty-nine percent (18/46) of samples from Kachin State, 62% (115/186) from Chin State, and 80% (40/50) from Karen State yielded acceptable copy number estimates. Copy number ranged from 0.72 to 3.70. 11.6% (19/173, 95%CI = 2.7-20.4) of samples had >1.5 copies and 4.6% (8/173) had >2 copies. The proportion of isolates with copy number >1.5 (95% CI) was 9.6% in Kachin State, 11.1% in Chin State, and 17.5% in Karen State (Table 2 and Figure 2). Isolates from population-screening participants were more likely than those from febrile clinical patients to have elevated *pfmdr1* copy number (OR 3.7, 95% CI 1.1-12.5). There was no difference in *pfmdr1* amplification between participants who did and did not report taking anti-malarial treatment in the previous eight weeks.

**Table 2:** ***Pfmdr1 *copy number (CN) among subclinical and clinical isolates from three regions of Myanmar 2008–2009**

	***pfmdr1 *CN**				**% CN >1.5**	
	**1**	**2**	**3**	**4**	**(95% CI)**^**a**^	**OR (95% CI)**^**a**^
**Karen State (n = 40)**	**33**	**6**	**0**	**1**	**17.5 (0–54.3)**	
Clinic	21	0	0	0	0.0	
Screening	12	6	0	1	35.0	n/a
**Kachin State (n = 18)**	**16**	**2**	**0**	**0**	**9.6 (0–31.6)**	
Clinic	5	1	0	0	16.7	
Screening	11	1	0	0	8.3	0.5 (0.0–7.8)^b^
**Chin-Mizoram (n = 115)**	**104**	**11**	**0**	**0**	**11.1 (0–32.1)**	
Clinic	76	6	0	0	7.3	
Screening	28	5	0	0	15.2	2.3 (1.1-4.7)^b^
**Screening vs clinicalTotal (n = 173)**	**153**	**19**	**0**	**1**	**11.6 (2.7-20.4)**	
Clinic	102	7	0	0	6.4	
Screening	51	12	0	1	20.3	3.7 (1.1-12.5)^b^
**Anti-malarial treatment Total (n = 168)**	**148**	**20**	**0**	**1**		
No treatment in past 8 weeks	117	17	0	1	12.7	
Treatment in past 8 wks	31	3	0	0	8.8	0.7 (0.1-3.1)^c^

^a^CIs adjusted for clustering within study site (n = 13).

^b^OR = odds of *pfmdr1* amplification among screening participants/odds of amplification among febrile clinical patients.

^c^ OR = odds among participants reporting treatment in past 8 weeks/odds among those not reporting treatment in previous eight weeks.

### *Pf* DNA concentration and precision of *pfmdr1* copy number determination

Of all CN estimates calculated, including repeat assays, 83 of 157 asymptomatic screening isolates (55%) and 23 of 133 clinic isolates (17%) had range:mean ratios greater than 0.5 and/or mean *Pf* b-tubulin Ct values greater than 34, indicating a *Pf* DNA concentration insufficient to estimate *pfmdr1* CN. Precision of *pfmdr1* copy number decreased with decreasing *P. falciparum* DNA concentration (Figure 2). The mean *P. falciparum* DNA concentration was lower for screening samples than for clinical samples and the precision of copy number estimates was lower overall for screening samples (Figure 2).

### *pfcrt* wild-type and K76T alleles

Multiplex PCR detected the wild-type *pfcrt* allele in 11 of 290 isolates (Table 3). Eight of 186 isolates from surveillance sites in Chin State carried the sensitive allele, as did three of 46 from sites in Kachin State; the sensitive allele was not identified in any of the 57 samples from Karen State. Twenty isolates failed to amplify either the sensitive or the K76T allele. Prevalence of the wild-type *pfcrt* allele was similar in isolates from active screening participants and febrile clinical patients, and did not vary by report of anti-malarial treatment in the previous eight weeks.

**Table 3:** ***Pfcrt *genotypes among subclinical and clinical isolates from three regions of Myanmar 2008-2009**

	**No. (%)**		
	**Resistant**^**a**^	**WT K76**	**OR (95% CI)**^**a**^
**Karen State (n = 58)**	**58 (100)**	**0 (0.0)**	
Clinic	27 (100)	0 (0.0)	
Screening	31 (100)	0 (0.0)	n/a
**Kachin State (n = 46)**	**43 (93.5)**	**3 (6.5)**	
Clinic	12 (100)	0 (0)	
Screening	31 (91.2)	3 (8.8)	n/a
**Chin-Mizoram (n = 186)**	**178 (95.7)**	**8 (4.3)**	
Clinic	88 (93.6)	6 (6.4)	
Screening	90 (97.8)	2 (2.2)	0.3 (0.1-1.7)
**Screening vs clinic Total**^**b**^**(n = 232)**	**221 (95.3)**	**11 (4.7)**	
Clinic	100 (94.3)	6 (5.6)	
Screening	121 (96.0)	5 (4.0)	0.7 (0.2-2.2)
**Anti-malarial treatment Total**^**c**^**(n = 232)**	**221 (95.3)**	**11 (4.7)**	
No treatment in past 8 weeks	174 (95.6)	8 (4.4)	
Treatment in past 8 weeks	47 (94.0)	3 (6.0)	1.4 (0.4-5.0)

^a^Resistant genotypes: 259 amplified K76T; n = 20 isolates that failed to amplify either K76T or wild type K76 were assumed to harbour a resistant genotype (eg SVMNT). Dropping these 20 observations from the analysis does not qualitatively modify the results.

^b^Standard errors corrected for clustering within village cluster (n = 13).

^c^Totals exclude Karen State given apparent fixation (100%) of the K76T allele.

Percentages may not sum to 100 due to rounding.

#### Systematic review

**Figure 3:**
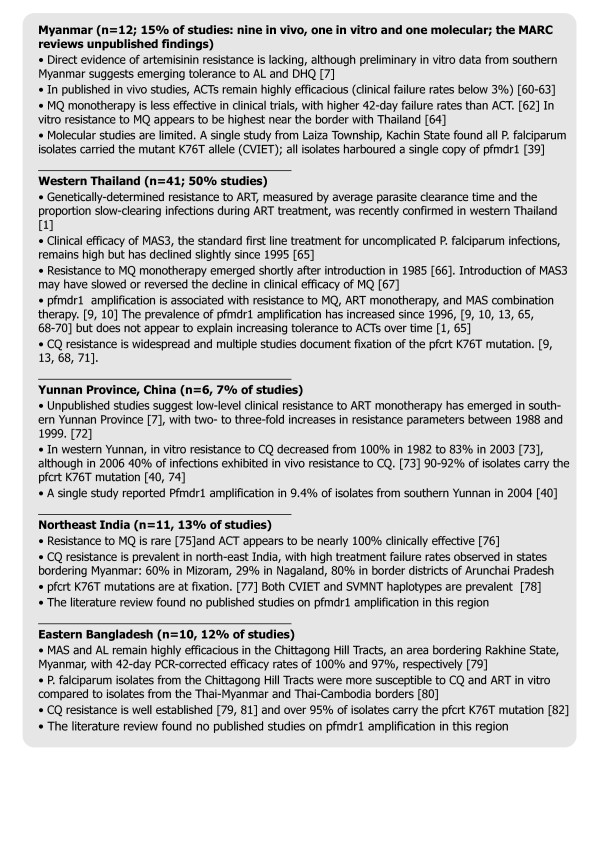
**Systematic review of *in vivo*, *in vitro*, and molecular resistance studies in Myanmar and neighbouring countries, 1996–2009[**[1,7,9,10,13,39,40,60-82]**].**

See Figure 3 and Additional file 1 for results of the brief systematic review of *in vivo*, *in vitro* and molecular resistance studies. Half of the 82 resistance studies meeting inclusion criteria were conducted in western Thailand, including all but two studies of *pfmdr1* CN. One study of *pfmdr1* CN from Myanmar was identified, conducted in Laiza in 2009 (Figure 1B). Locations of *in vivo*, *in vitro* and molecular studies did not overlap with areas of instability, indicated forcibly displaced villages (red shading) on the map in Figure 1.

## Discussion

This study demonstrates the feasibility of incorporating a network of health workers affiliated with community-based organizations into strategies for molecular surveillance of malaria resistance in remote and unstable areas of Myanmar, including priority Tier 1 and Tier 2 areas at high risk for the spread of delayed parasite clearance. This surveillance network can accelerate the assessment of artemisinin resistance and delayed parasite clearance once genetic markers are identified. Myanmar’s ecologic, ethnic and political diversity, coupled with patchwork access to malaria control services and quality anti-malarials creates a mosaic of selective drug pressure, and this study provides a preliminary glimpse of patterns emerging over time and place in remote areas of the country, including areas directly across the border from western Thailand where delayed parasite clearance was recently confirmed. The prevalence of *pfmdr1* amplification was relatively low (12-19%) across the three border regions included in the current study; and only a single isolate had a CN of three or more. Results from Karen State contrast with recent studies documenting elevated CN in at least 40% of isolates [9] with a mean CN of 2.9 (SE 1.4) [39] among refugee, migrant and cross-border populations in Thailand (Figure 1). Findings from Kachin State contrast with a recent study in Laiza that found no *pfmdr1* amplification in 171 *P. falciparum* isolates [40,41], but is consistent with a study conducted in 2004 in nearby counties of Yunnan province [42]. The systematic review failed to identify previous studies of *pfmdr1* CN along the borders with India and Bangladesh to compare to the 11% prevalence estimated for that region.

Multiple factors likely contribute to the observed variation in *pfmdr1* amplification documented within and between populations living in Myanmar and across its borders in neighbouring countries. Little is known about host response in populations in Myanmar [24], but host immunity is likely to be relatively more robust given higher transmission intensity compared to western Thailand and Yunnan, where migrants from Myanmar account for the majority of infections [19,43-46]. Selective drug pressure also varies substantially due to differences in access to quality diagnosis and treatment. Populations included in the present study live in areas that government and international NGO health services largely fail to reach, and community based organizations are unique providers of malaria control interventions. The low prevalence of *pfmdr1* amplification in Karen study sites may be due in part to these malaria programmes [17] that for 10 years have provided directly observed mefloquine-artesunate that may modify selective pressure for *pfmdr1* amplification [47].

The *pfcrt* K76T mutation is a highly predictive marker of CQ resistance [48]. Replacement of K76T mutants with wild-type parasites following withdrawal of drug pressure has forecast the return of CQ clinical efficacy [49,50] and some authors have proposed tracking this mutation to identify populations in which CQ could be used as partner drugs in ACT [51]. The very low prevalence of wild-type *pfcrt* in the present study is consistent with data from nearby areas and suggests ongoing selective pressure for CQ resistance. Although decades have passed since CQ was officially recommended by Myanmar or any of its neighbours for the treatment of *P. falciparum* infection, CQ remains the treatment of choice for *P. vivax* and presumptive malaria. The use of RDTs capable of identifying both *P. falciparum* and *P. vivax* species may modestly diminish this selective pressure, but tracking the prevalence of wild-type *pfcrt* is likely to remain a low priority for resistance surveillance for the next several years. However, *pfcrt* could become an important genetic marker if the recommended treatment of *P. vivax* changes from CQ to ACT in response to the recent emergence of CQ-resistant *P. vivax* in western Thailand [52,53].

Consistent with the *a priori* hypothesis, the prevalence of elevated *pfmdr1* CN was higher among isolates obtained from active screening participants than from isolates obtained from febrile clinical patients. Resistance surveillance currently relies on samples obtained from clinically symptomatic malaria patients; containment efforts may need to more aggressively target subclinical infections that could serve as a reservoir for the spread of drug resistance if future studies validate an association between subclinical infection and resistance alleles. However, there are several reasons to approach this finding with caution. First, to the authors’ knowledge this is the first study to evaluate *pfmdr1* CN in a predominantly asymptomatic population. Zhong *et al.*[54] found no difference between symptomatic and asymptomatic study volunteers in Kenya in the frequency of *pfmdr1* point mutations, or of the K76T mutation of *pfcrt.* Second, consistent with Zhong *et al.* the current study found no difference in K76T mutation prevalence between screening and clinical participants, though prevalence of wild-type *pfcrt* was low in each region and results may be confounded by ongoing CQ drug pressure as described above. Third, a robust empirically derived conceptual model is lacking to adequately capture the complex interactions between transmission intensity, host immunity, drug pressure, fitness cost, compensatory mutations, clonality of infection, de-amplification [27,55] and other factors expected to influence the relative prevalence of resistant and wild-type parasites in clinical and asymptomatic infections. Clonality of infection is likely to play an important role, as described above, but the prevalence of monoclonal infection in Myanmar is poorly quantified. A single study from central Myanmar documented mono-infection in 32% of clinical isolates [56], but the relevance of these data to asymptomatic populations in border regions is unclear. The final reason to interpret with caution the association between subclinical infection and genetic resistance is the high variance of *pfmdr1* CN estimates found among screening participants that led to the exclusion of 55% of samples. Future studies are needed to validate the accuracy of *pfmdr1* CN estimates based on filter-paper blood samples collected from subclinical populations. Despite these caveats, findings presented here highlight the need to conduct additional studies on the contribution of subclinical infection to the epidemiology of drug-resistant malaria.

Although mosquitoes and migrating humans frequently carry parasites across international boundaries, it is not clear whether findings from neighbouring countries should be extrapolated to populations living in Myanmar. Individuals seeking care from border clinics by definition have access to diagnostic and treatment protocols and other resources of neighbouring countries that may differ substantially from those available in remote and conflict-affected areas of Myanmar. Additional studies are necessary to determine the appropriate geographic scale for monitoring the spread of drug resistance.

Systematic review of *in vivo*, *in vitro* and molecular resistance studies from Myanmar and its border regions identified relatively few data from populations living inside the country, particularly in Tier 1 and 2 areas of highest priority for resistance containment. The paucity of available information is consistent with output from the under-developed health research capacity in the country as a whole: Myanmar ranks 218^th^ out of 224 countries in number of publications in medicine per capita (0.4 per 100,000 people) [57]. Armed conflict and large-scale population displacement are established causes of disruptions in health services and disease surveillance [58], and can result in “stability bias”, defined as the “systematic under-sampling of populations and health threats in contexts of conflict and instability” [59]. The lack of overlap between previous resistance study locations and displaced populations in Myanmar documented in the present study suggests that stability-bias may contribute to within-country distribution of evidence available for resistance surveillance. The analysis presented here highlights areas with documented population displacement, but does not capture all conflict and human rights violations experienced by other communities. For example, approximately 92% of households in Chin State may experience forced labour [31] and other human rights violations that have been associated with prevalent malaria infection [32]. The instability of sites participating in this study was tragically validated by violent events since data collection completed in 2009: one Karen site and all Kachin sites were displaced due to military attacks. Some health workers have continued to implement disease control interventions in relocated areas; and they remain willing to participate in resistance surveillance activities. One community-based organization is establishing a site to monitor parasite clearance time.

There were several important limitations to this study. Twenty *P. falciparum* isolates did not amplify either wild type or K76T alleles and it is not possible to exclude the presence of other rare resistance alleles such as SVMNK that were not assessed. None of these twenty isolates produced valid copy number estimates, and they do not modify our primary conclusions that *pfmdr1* amplification may be more common among subclinical infections, and that that wild type *pfcrt* remains rare in these three regions of Myanmar.

The precision of *pfmdr1* CN estimates determined from subclinical infections was low for 44% of isolates, and 16% of isolates had DNA concentrations below the lower limit of reliable copy number determination, even after four-fold DNA concentration with glycogen-acetate precipitation. As noted above, future studies are necessary to validate the use of filter-paper samples to estimate gene CN from low-parasitaemia isolates such as those collected from subclinical infections. This study was not designed to elucidate the mechanisms or relative contributions of factors influencing the prevalence of resistant parasites in subclinical populations. For example, clonality of infection was not determined and parasite DNA from prior infections was unavailable to distinguish re-infection from recrudescence.

The cross-sectional design limits causal inference, and findings related to measures of association should be considered preliminary, as noted above. Study participants lived in areas where quality malaria control services were ongoing for three to 8 years, and findings may not apply to intervention-naïve populations. Samples were collected in 2008–2009 and may not reflect the prevalence of resistance markers in 2012. For example, areas of western Thailand north of Maesot, immediately across the border from one of the present study sites, experienced a sharp rise in the proportion of slow-clearing parasites between 2008–10 [1,2].

The number of *P. falciparum-*positive isolates (290) was lower than anticipated by the study design (540) despite screening 4,591 villagers and 988 febrile clinical patients. Logistical constraints delayed data collection in Karen and Kachin areas until the lower-transmission, dry-season months of February and March, with extended clinical sampling through June. The study was conducted in areas of active malaria control, and the success of these programmes likely contributed to the low prevalence of *P. falciparum* and low RDT positivity rates. The smaller than anticipated number of *P. falciparum* positive samples limited the precision of estimates of genotype prevalence in subclinical and clinical infections, and poor statistical power precluded comparisons across geographic regions. Nevertheless, the number of isolates available for genotyping compares favourably to studies conducted in more stable areas, and data from individuals with subclinical *P. falciparum* infections is unique among published studies from this region.

## Conclusions

Networks of community-based health organizations can and should contribute to molecular surveillance of anti-malarial drug resistance in remote and unstable areas of Myanmar, including priority Tier 1 and 2 areas at greatest risk for spread of delayed parasite clearance. Desiccated filter paper facilitates accurate estimation of *pfmdr1* CN among febrile clinical isolates and extends the potential geographic range of genetic resistance surveillance, but further study is necessary to validate the use of filter paper in the setting of subclinical infections with low parasite DNA concentrations. The increased risk of *pfmdr1* amplification observed among active screening participants hints at a plausible and potentially important contribution of subclinical infection to resistance transmission, but further study is necessary to elucidate the epidemiology of drug resistance among predominantly asymptomatic populations. The consistently low prevalence of wild-type *pfcrt* alleles, documented in this and other studies, is consistent with persistent CQ drug pressure that is unlikely to diminish until treatment protocols specify CQ-free regimens for *P. vivax* infections. A systematic review of resistance studies revealed a dearth of data from Myanmar, particularly from areas recently experiencing armed conflict and forced displacement. Marginal populations, such as those participating in this study, should be represented in future resistance surveillance efforts, particularly once genetic markers of delayed parasite clearance are identified. Failure to include these at-risk areas may compromize global efforts to monitor and contain the spread of resistance.

## Abbreviations

ACPR: Acceptable Clinical and Parasitological Result; ACT: Artemisinin combination therapy; AL: Artemether-lumefantrine; ART: Artemisinin (eg: artesunate, artemether); CN: Copy number; CQ: Chloroquine; DHP: Dihydroartemisinin-piperaquine; MAS: Mefloquine artesunate; MQ: Mefloquine; pfcrt: *Plasmodium falciparum* CQ resistance transporter; pfmdr1: *Plasmodium falciparum* multidrug resistance protein (*pfmdr1*).

## Competing interests

All authors declare that they have no competing interests.

## Authors’ contributions

TB carried out the laboratory work, conducted data analysis and literature review, and drafted the manuscript. LS assisted with conception and design of the field study, oversaw fieldwork, takes responsibility for the integrity of the field data, and edited the manuscript; EK and KS supervised fieldwork in Karen State and Kachin State, respectively, and critically reviewed the manuscript; DS assisted with the design of the study, supervised PCR laboratory work and critically reviewed the manuscript; TL and CB assisted with the conception and design of the field study and edited the manuscript; AR conceived and designed the study, conducted data analysis and literature review and drafted the manuscript. All authors read and approved the final manuscript.

## Supplementary Material

Additional file 1 **Brown.** Supplementary Appendix 1.docx. Peer reviewedcross-sectional studies reporting prevalence estimates for *pfmdr1* copy number or pfcrt K76T haplotypes in Burma and adjacent areas of neighboring countries, 1996-2009. Summarizes cross-sectional molecular studies of antimalarial drug resistance included on the map in Figure 1. Click here for file
