# Supplementary material for: Molecular surveillance for drug-resistant Plasmodium falciparum in clinical and subclinical populations from three border regions of Burma/Myanmar: cross-sectional data and a systematic review of resistance studies
Source: Malar J. 2012 Sep 19;11:333. doi: 10.1186/1475-2875-11-333 (PMC3518194; doi:10.1186/1475-2875-11-333)
Supplement: Additional file 1 — Brown. Supplementary Appendix 1.docx. Peer reviewedcross-sectional studies reporting prevalence estimates for pfmdr1 copy number or pfcrt K76T haplotypes in Burma and adjacent areas of neighboring countries, 1996-2009. Summarizes cross-sectional molecular studies of antimalarial drug resistance included on the map in Figure 1. [file 1475-2875-11-333-S1.docx]

**Supplementary Table S1. Peer-reviewed cross-sectional studies reporting prevalence estimates for *pfmdr1* copy number or *pfcrt* K76T haplotypes in Burma and adjacent areas of neighboring countries, 1996-2009.**

|  | **First author** | **Publication year** | **Sample collection year** | **Study location(s) of interest** | **Sample size**  **(successfully genotypted)** | **% isolates with *pfmdr1* copy number >1** | **% isolates with K76T *pfcrt* haplotype** |
| --- | --- | --- | --- | --- | --- | --- | --- |
| 1 | Carrara | 2009 | 1996, 2006 | Tak Province, Northwestern Thailand | (a) 1996: 40  (b) 2006: 45 | 1996: 30%  2006: 53% | n/a |
| 2 | Price | 2004 | 2002 | Tak Province, Northwestern Thailand | 532 | 54% | 100% |
| 3 | Nelson | 2005 | 2002 | Sangkhlaburi District, Kanchanaburi Province, Western Thailand | 52 | 9.8% | n/a |
| 4 | Price | 2006 | 2002 | Tak Province, Northwestern Thailand | 92 | 48% | n/a |
| 5 | Congpuong | 2005 | 2003 | Kanchanburi, Tak, Ranong, and Chiang Mai Provinces | 280 | n/a | 99% |
| 6 | Chaijaroenkul | 2011 | 2003 | Kanchanaburi, Tak, Ratchaburi and Ranong Provinces | 95 | n/a | 95% |
| 7 | Setthaudom | 2011 | 2005 | Thai-Burma border | 68% | n/a | 98% |
| 8 | Chaijaroenkul | 2010 | 2007 | Tak Province, Northwestern Thailand | 26 | 36% | 96% |
| 9 | Imwong | 2010 | 2008 | Tak Province, Northwestern Thailand | *pfmdr1*: 35  *pfcrt*: 37 | 46% | 100% |
| 10 | Muhamad | 2011 | 2009 | Tak Province, Northwestern Thailand | *pfmdr1*: 117  *pfcrt*: 134 | 40.2% | 100% |
| 11 | Phompradit | 2011 | 2009 | Thai-Burma border | 58 | 57% | n/a |
| 12 | Brown | 2012 | 2009 | Karen State, Kachin State, and China State, Burma; Mizoram State, India | *pfmdr*: 163  *pfcrt*: 208 | 12% | 94.7% |
| 13 | Yang, Z | 2007 | 2004 | Gengma County and Mengla County, Yunnan Province, China | 99 | 9.4% | 90% |
| 14 | Zhang, GQ | 2009 | 2007 | Yunnan Province, China | 74 | n/a | 92% |
| 15 | Yang, Z | 2011 | 2009 | Laiza, Kachin State | 117 | 0% | 100% |
| 16 | van den Broek | 2004 | 2002 | Chittagong Hill Tracts, Bangladesh | 187 | n/a | 94% |
| 17 | Mittra | 2006 | 2001, 2004 | Assam State, Northeastern India | 40 | n/a | 100% |
| 18 | Kawai | 2011 | 2004 | Rangmati, Bangladesh | 45 | n/1 | 88% |
| 19 | Keen | 2007 | 2006 | Assam State, Northeastern India | 66 | n/a | 97% |
| 20 | Awasthi | 2011 | 2006 | Mizoram, Meghalaya States, Northeastern India | Mizoram: 30  Meghalaya: 30 | n/a | 100% |
